# Hybrid intelligent systems for liver disease prediction: a demographic-aware machine learning framework

**DOI:** 10.3389/fmed.2025.1728061

**Published:** 2026-01-12

**Authors:** Ekta Saraf, Mao Yang, Ramalingam Sakthivel, Yiquan Zhang, Santosh Chokkakula, Yu Kong, Vykunta Alekya, Bommireddy Naveen, Bing Yang

**Affiliations:** 1 School of Computer Science and Engineering, Vellore Institute of Technology, Chennai, India; 2 Department of Invasive Interventional Therapy, Tianjin Cancer Hospital, Airport Hospital, Tianjin, China; 3 School of Pharmacy, Tianjin Medical University, Tianjin, China; 4 Department of Microbiology, Chungbuk National University College of Medicine and Medical Research Institute, Cheongju, Republic of Korea; 5 Department of Cell Biology, College of Basic Medical Sciences, Tianjin Medical University, Tianjin, China; 6 SVU Health Centre, Sri Venkateswara University, Tirupati, Andhra Pradesh, India; 7 Department of Chemical and Biological Engineering, Gachon University, Seongnam, Gyeonggi, Republic of Korea; 8 Department of Public Health, International School, Krirk University, Bangkok, Thailand

**Keywords:** gender-age dynamic machine learning, hybrid intelligent, liver disease prediction, machine learning based classification, random forest

## Abstract

**Background:**

Liver disease remains a major global health burden, often progressing undetected until advanced stages. Traditional diagnostic approaches, while accurate, are invasive, costly, and limited in accessibility.

**Objective:**

To address these challenges, we propose a hybrid intelligent framework that integrates demographic segmentation with advanced machine learning for the early detection of liver disease.

**Results:**

Two datasets were employed, including the Indian Liver Patient Dataset (ILPD, *n* = 583) and a large-scale dataset (*n* = 29,787). Patients were stratified by age and gender into six groups, enabling segment-specific model development. Sixteen algorithms, including Random Forest, Support Vector Machines, XGBoost, and LightGBM, were evaluated using recursive feature elimination, resampling techniques, and Bayesian hyperparameter optimization. Segment-specific best models were integrated into a hybrid system through dynamic selection and ensemble strategies. The framework achieved 94.2% accuracy on ILPD and 99.8% on the large dataset, with consistent improvements across demographic groups. Feature analysis revealed distinct biomarker importance by age and gender, underscoring the need for tailored diagnostic approaches..

**Conclusion:**

By combining demographic awareness, hybrid learning, and interpretability, this study offers a scalable, non-invasive, and clinically relevant tool for early detection of liver diseases, advancing personalized and accessible healthcare.

## Introduction

Liver disease is a major global health burden, often progressing silently until advanced stages (1–5). Responsible for over 2 million deaths annually, it remains difficult to detect early, with nearly 70% of cases diagnosed too late for effective intervention. Although the liver performs vital functions, including detoxification, protein synthesis, and metabolic regulation, even minor imbalances can have severe consequences. Conventional diagnostics such as biopsies, imaging, and biochemical testing, while accurate, are invasive, costly, and often unavailable in low-resource settings, widening healthcare disparities.

The rise of artificial intelligence (AI) and machine learning (ML) has opened new opportunities to overcome these diagnostic challenges. By leveraging complex, multidimensional biomedical datasets, ML can reveal hidden patterns that traditional methods often overlook, enabling earlier and more accurate disease prediction (6, 7). In recent years, numerous ML-based systems have been developed for disease diagnosis, ranging from cardiovascular disorders to chronic liver conditions. For example, hybrid intelligent frameworks that combine multiple algorithms have demonstrated superior accuracy and interpretability compared to single models (8–14). Seera et al. (12) integrated Fuzzy Min-Max Neural Networks with decision trees and Random Forests, improving robustness and adaptability for noisy medical datasets. Similarly, Haq AU et al. (13) applied feature selection strategies such as Relief and LASSO alongside multiple classifiers, achieving enhanced accuracy in heart disease prediction. These studies highlight the potential of hybrid and ensemble systems for clinical decision support.

In the domain of liver disease, several machine learning models have shown promise. Rahman et al. (15) compared multiple algorithms on the Indian Liver Patient Dataset (ILPD), finding Logistic Regression to be most effective, while Naive Bayes underperformed. Theerthagiri et al. (4) introduced Histogram-based Gradient Boosting with recursive feature selection, which significantly improved predictive accuracy. Khaled et al. (16), using a large dataset of over 32,000 records, demonstrated that Random Forests achieved over 97% accuracy in early liver disease detection. Ganie et al. (17) advanced this field further by applying boosting techniques such as XGBoost, LightGBM, and CatBoost, which enhanced performance across multiple benchmark datasets. Collectively, these works underscore the growing role of ML in liver disease diagnosis.

Recent advancements in explainable and ensemble-based liver disease prediction have also contributed significantly to this domain. Mamun et al. (18) proposed an explainability-enhanced diagnostic approach using tree selection and a stacking ensemble of Random Forest models, demonstrating the importance of combining model accuracy with transparent decision paths. Similarly, Chowdhury et al. (19) introduced a hybrid ensemble framework that integrates artificial neural networks with advanced explainable AI to identify optimal diagnostic features and improve clinical interpretability. In addition, Zhu et al. (3) applied interpretable machine-learning techniques to predict metabolic dysfunction–associated steatotic liver disease (MASLD), emphasizing the role of demographic and biochemical variables in disease risk. While these studies highlight the value of explainable and ensemble methods, they do not consider demographic heterogeneity. Our work extends this direction by incorporating explicit age–gender segmentation and dynamic hybrid model selection, enabling subgroup-specific interpretability and improving fairness across diverse patient cohorts.

More recent innovations incorporate hybrid deep learning and optimization strategies to refine prediction models. Behera et al. (20) combined Support Vector Machines with Particle Swarm Optimization for fine-tuned classification. Islam et al. (21) optimized ML algorithms using Tree-Structured Parzen Estimators, achieving 95.8% accuracy with Extra Tree Classifiers on ILPD. Ampavathi et al. (22) extended the predictive scope to multiple diseases by integrating Deep Belief Networks and Recurrent Neural Networks with advanced metaheuristic optimization. These approaches emphasize the value of combining ML with optimization techniques to achieve higher accuracy and generalizability.

Despite these advances, critical gaps remain. Most existing models adopt generalized approaches that fail to account for demographic differences such as age and gender, even though disease presentation and biomarker relevance vary substantially across populations. For instance, the predictive weight of albumin or bilirubin may differ between elderly males and younger females. Few studies have systematically explored demographic-aware segmentation, dynamic model selection, or the contextual interpretation of biomarkers across groups (23, 24). Moreover, algorithmic complexity is often prioritized over clinical interpretability, limiting real-world adoption.

To address these limitations, this study proposes a demographic-aware hybrid machine learning framework for liver disease prediction. The framework explicitly segments patients by age and gender to create homogeneous subgroups, applies recursive feature elimination to identify key biomarkers, and employs resampling techniques such as SMOTE to address class imbalance. Dynamic model selection ensures that the most suitable algorithm is deployed for each subgroup, while Bayesian hyperparameter optimization maximizes performance. By validating the framework on both a benchmark dataset (ILPD, *n* = 583) and a large real-world dataset (*n* = 29,787), this work establishes cross-dataset robustness. To further validate the necessity of segmentation, we additionally trained a unified baseline model without demographic stratification.

## Methods

### Study design and objective

**Figure 1:**
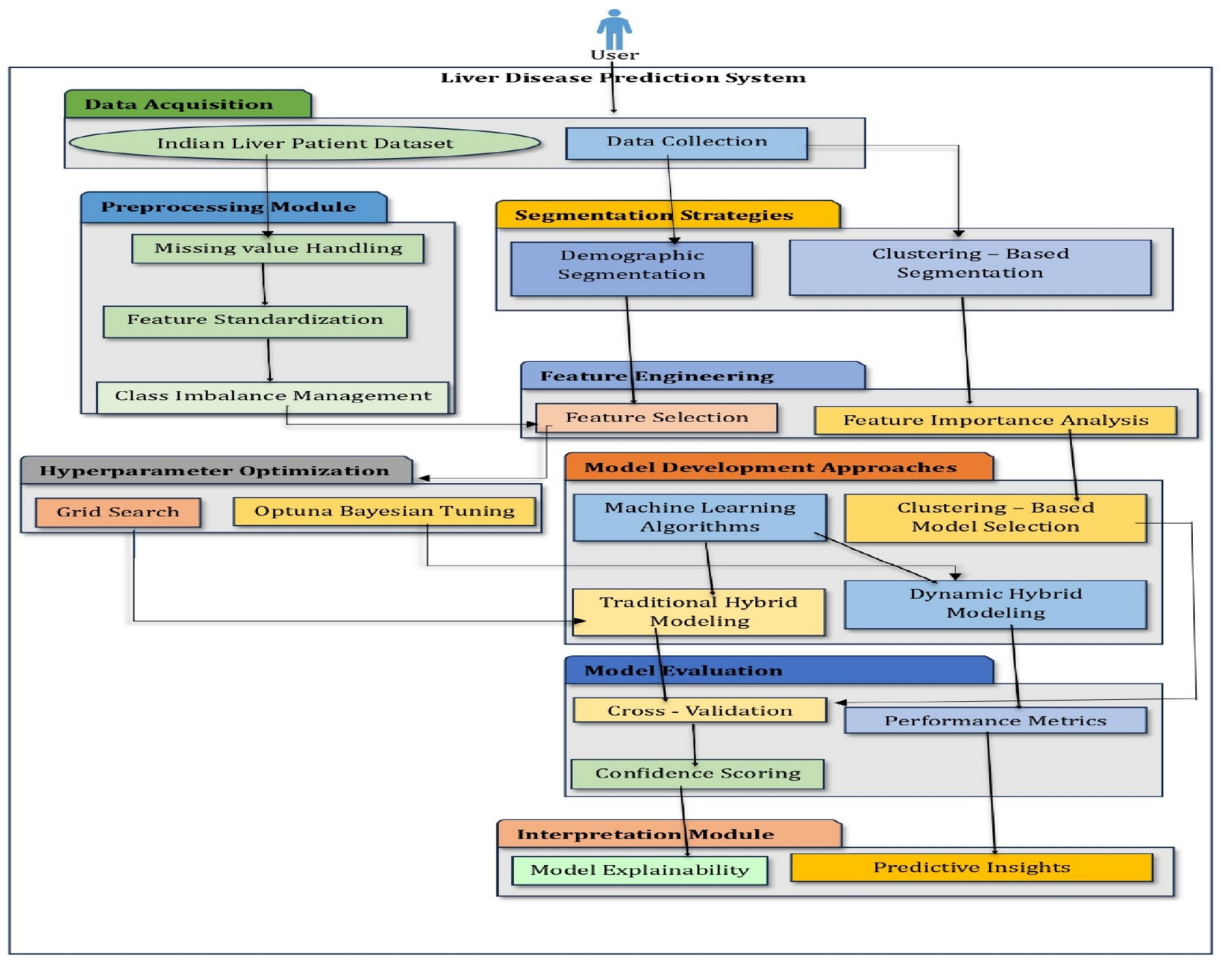
Architecture of a hybrid intelligent system.

This study aimed to develop a gender and age-aware hybrid framework for liver disease prediction that integrates demographic segmentation with ensemble learning techniques. The primary goal was to enhance predictive accuracy while maintaining interpretability and clinical relevance. The overall workflow of the proposed system, illustrated in Figure 1, involves sequential steps including data preprocessing, demographic segmentation, segment-specific model development, and integration into a hybrid prediction framework.

### Datasets

Two datasets were employed to evaluate the proposed methodology. The Indian Liver Patient Dataset (ILPD) comprises 583 patient records, each with 11 features, including age, gender, and nine biochemical markers such as Total Bilirubin, Direct Bilirubin, Alkaline Phosphatase, Alanine Aminotransferase, Aspartate Aminotransferase, Total Proteins, Albumin, and Globulin. This dataset was sourced from the UCI Machine Learning Repository. The second largest dataset, the Extended Liver Dataset (*n* = 29,787), has 21,917 samples labeled as diseased and 8,774 as healthy. The dataset was sourced from the publicly available Kaggle liver disease repository and includes identical features to ILPD. To ensure consistent preprocessing, missing values were imputed using median values computed globally for the unified model and segment-specific medians for the demographic-aware framework. This dataset was used to evaluate the robustness and scalability of the proposed approach.

Formally, the datasets can be represented as:


$$\;\;D={\left\{(x_{i},y_{i})\right\}}_{i=1}^{N}\;\;\text{or}\;\;x_{i}\in R^{d},y_{i}\in \{0,1\}\;$$ (1)

where $x_{i}\in R^{d}$ is the feature vector of the *i*-th patient and $y_{i}\in \{0,1\}$ is the binary label (0: no disease, 1: disease).

### Data preprocessing

Data preprocessing was conducted to ensure quality, consistency, and suitability for model training. Missing values were addressed using segment-specific median imputation, which preserves demographic consistency,


$$M(x_{i})=x_{i}^{\prime },x_{i}[j]=\text{median}(x_{k}[j])\text{for}\;k\in \text{segment}(i)$$ (2)

Feature scaling was performed using Z-score normalization independently within each demographic segment,


$$S(x_{i}^{\prime })=x_{i}^{\prime \prime }$$ (3)

$$\;x_{i}^{\prime \prime }[j]=\frac{x\prime [j]-\mu_{j}}{\sigma_{j}}$$

where $\mu_{j}$ and $\sigma_{j}$ denote the mean and standard deviation of the j-th feature within the segment.

### Demographic segmentation

Patients were categorized into six biologically relevant groups based on age (Young: <35, Middle-aged: 35–55, Elderly: >55) and gender (Male, Female). This segmentation aimed to form homogeneous subsets for model development, reflecting variations in disease manifestation across demographics.

### Handling class imbalance

Class imbalance within each segment was addressed using adaptive resampling strategies. Small segments with fewer than 50 samples were balanced using Borderline-SMOTE combined with conditional undersampling. Medium segments (50–100 samples) were processed using SMOTETomek, while larger segments employed standard SMOTE when the minority class represented less than 90% of the majority class. All resampling methods were applied only on the training data after the train-test split, and never on the test data, to avoid information leakage. The resampling process can be formalized as,


$$(X\prime \prime ,Y)=(X_{\text{resampled}},Y_{\text{resampled}})\;$$ (4)

### Data splitting

Each segment was split into training and testing sets using an 80:20 stratified split, preserving class distributions across both sets. To ensure robust evaluation, stratified 5-fold cross-validation was used. Each fold maintains an approximate 80:20 ratio between training and testing data; however, unlike a fixed single split, the folds are created using different randomized subsets.

### Segment-specific model development

For each demographic segment, a range of machine learning models was evaluated, including linear classifiers, tree-based ensembles, kernel-based methods, and neural networks. Recursive Feature Elimination (RFE) was applied to each segment using the Extra Trees Classifier to select the most predictive features,


$$\;F_{t+1}=F_{t}\{f\in F_{t}|I(f)\leq \tau_{t}$$ (5)

RFE was selected as the primary feature selection technique due to its suitability for structured clinical datasets with correlated biomarkers. Compared with filter-based methods (e.g., mutual information, ReliefF) and embedded techniques (e.g., LASSO, ElasticNet), RFE provides the advantage of evaluating feature relevance in the context of the model rather than in isolation. This approach allows iterative removal of less informative features while preserving feature–interaction effects, which are known to influence clinical biomarkers. Unlike previous liver disease studies, RFE offered a balance of interpretability and computational efficiency, enabling consistent processing across all 16 evaluated classifiers. RFE and all hyperparameter tuning using Optuna were performed strictly within the training folds only, ensuring that no information from the test data was used during model development. Additionally, hyperparameter optimization was conducted using Optuna, a Bayesian optimization framework, with a Tree-Structured Parzen Estimator (TPE) sampler,


$$\;\theta^{*}=\arg\max_{\left\{\theta \in \Theta \right\}}P(M_{\theta })$$ (6)

To ensure transparency and reproducibility, the search space for major algorithms was defined using parameter ranges commonly recommended in prior machine-learning literature. For tree-based models such as Random Forest, Extra Trees, XGBoost, and LightGBM, the hyperparameters included:

- Number of estimators (50–500),
- Maximum depth (3–30),
- Learning rate for boosting models (0.001–0.3),
- Minimum samples split (2–20),
- Maximum features (sqrt, log2, or 0.3–1.0).

For linear and kernel-based models such as Logistic Regression and SVM, the hyperparameters included:

- Regularization strength C (0.01–10),
- Kernel type (linear, RBF, polynomial),
- Gamma range for RBF kernel (1e-4 to 1e-1).

Each Optuna study was executed for 50 optimization trials, with early pruning applied if no improvement occurred over 10 successive trials. All hyperparameter tuning was performed strictly within the training folds of the stratified 5-fold cross-validation to avoid information leakage. The approximate computational time per segment ranged between 3 and 11 min, depending on algorithm complexity and dataset size.

### Hybrid prediction framework

The best-performing models from each demographic segment were integrated into a hybrid predictive framework, allowing for dynamic model selection. To ensure that each demographic segment received an optimally tailored model, a structured decision framework was implemented. Candidate models within each segment were ranked using F1-score as the primary criterion due to class imbalance and clinical relevance of minimizing false negatives. In cases with similar F1-scores (difference <0.01), other metrics and computational efficiency are used as a secondary comparator. This systematic prioritization ensured that the selected “best” model was both statistically optimal and clinically meaningful for each subgroup. Model performance was evaluated using accuracy, precision, recall, F1-score, and AUC-ROC, with particular emphasis on F1-score for its balanced representation of precision and recall. This hybrid framework enables the identification of segment-specific biomarkers and highlights differences in disease manifestation between age and gender groups, providing clinically meaningful predictions.

Model performance was assessed using the following standard metrics:


$$\text{Accuracy}:A=\frac{\mathrm{TP}+\mathrm{TN}}{\mathrm{TP}+\mathrm{TN}+\mathrm{FP}+\mathrm{FN}}$$

$$\text{Precision}:\text{Accuracy}:P=\frac{\mathrm{TP}}{\mathrm{TP}+\mathrm{FP}}$$

$$\text{Recall}:R=\frac{\mathrm{TP}}{\mathrm{TP}+\mathrm{FN}}$$

$$F1-\text{Score}:\;F1=\frac{2\mathrm{PR}}{P+R}$$

$$\mathrm{AUC}-\mathrm{ROC}:\text{Plot of}\;\mathrm{TPR}\;\text{and}\;\mathrm{FPR}\;\text{where}\;\mathrm{FPR}=\frac{\mathrm{FP}}{\mathrm{FP}+\mathrm{TN}}$$

Following the evaluation, three hybridization strategies were implemented:

Top N Model Selection: Ensembles were constructed from the top 1–4 models per segment.

Dynamic Model Selection: The single best-performing model was deployed for each segment.

Meta-Ensembling: Weighted aggregation of predictions from top-performing models.

### Segment-specific analysis

Performance was examined across both datasets. The extended dataset (29,787 patients) demonstrated consistent high performance across all demographic groups, confirming the clinical utility of segment-aware modeling. Optimal model selection varied by segment; tree-based ensemble methods (Extra Trees, XGBoost) performed best for male patients, whereas Quadratic Discriminant Analysis was superior for middle-aged females. Feature importance analysis revealed distinct patterns across demographics, with Alkaline Phosphatase consistently influential and Albumin particularly relevant for elderly and middle-aged females.

## Results

### Data characteristics and preprocessing outcomes

Two datasets of different scales were employed in this study. The Indian Liver Patient Dataset (ILPD) comprises 583 records, and the Extended Liver Dataset with 29,787 records. Both datasets included 11 attributes, covering demographic variables (age and gender) as well as nine biochemical markers relevant to liver function, such as Total Bilirubin, Direct Bilirubin, Alanine Aminotransferase (ALT), Aspartate Aminotransferase (AST), Alkaline Phosphatase (ALP), Albumin, Globulin, and Albumin/Globulin ratio. Data preprocessing incorporated several critical steps. Missing values were addressed through segment-specific median imputation, ensuring that imputed values were reflective of the age–gender subgroup rather than the overall distribution. This strategy preserved demographic-specific trends in biochemical markers, which could otherwise be masked by global imputation. Next, Z-score normalization was applied independently within each demographic group to standardize attributes while maintaining inter-segment variability. This scaling was crucial to ensure that no single biomarker disproportionately influenced model training.

**Figure 2:**
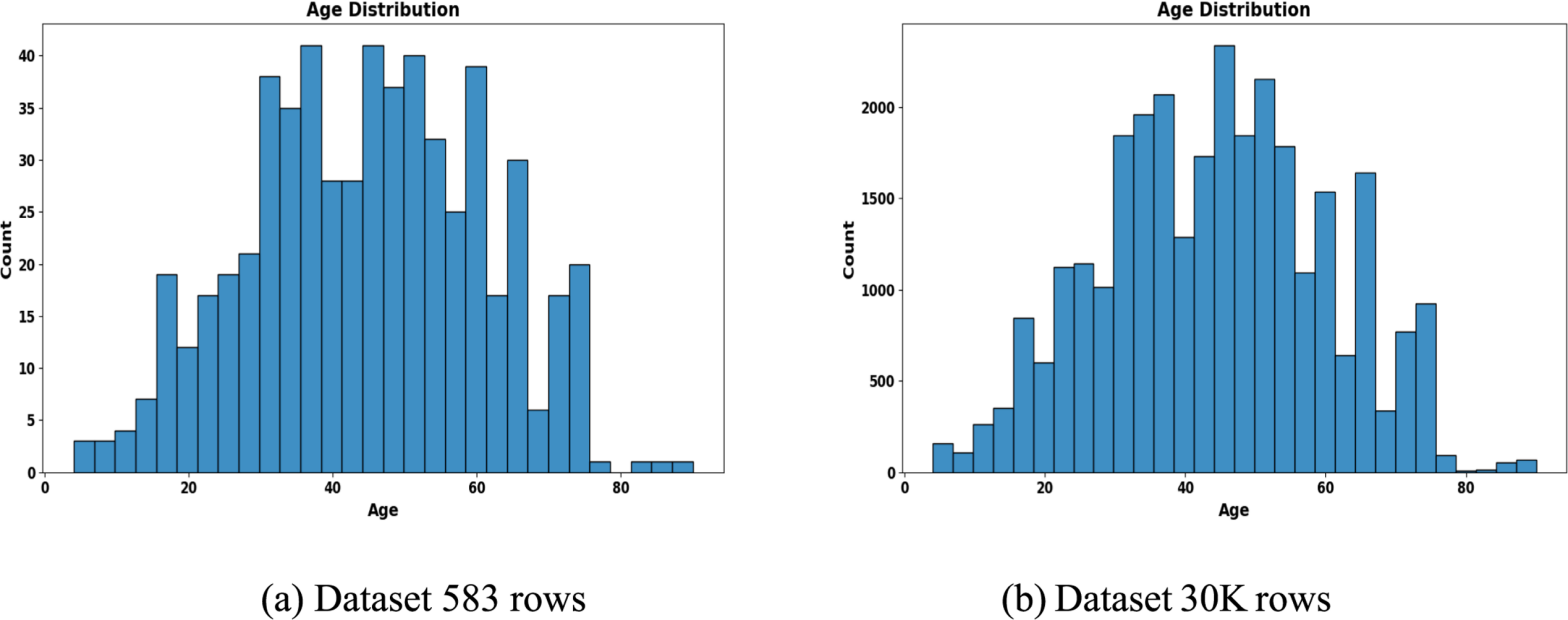
Age distribution histogram. **(a)** Dataset 583 rows **(b)** Dataset 30 K rows.

**Figure 3:**
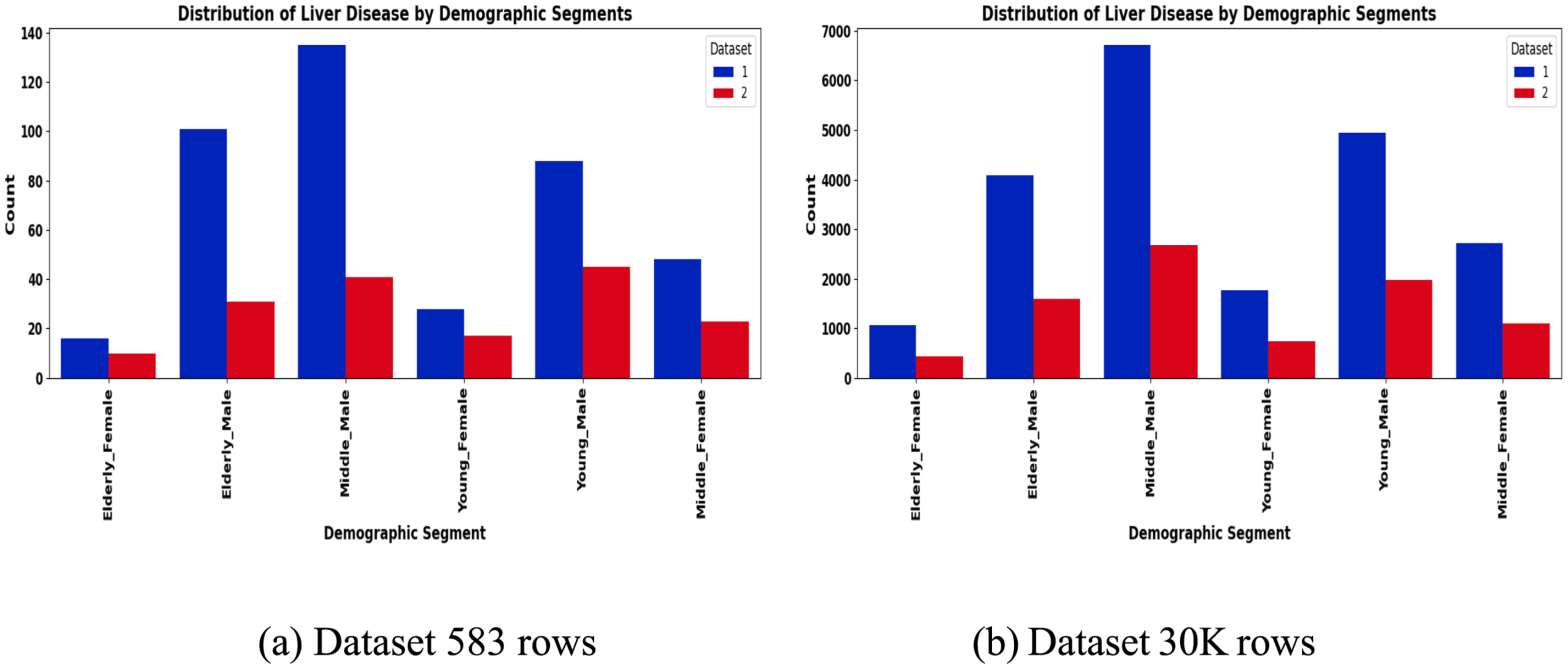
Liver disease prevalence by segment. **(a)** Dataset 583 rows **(b)** Dataset 30 K rows.

To address class imbalance, different resampling methods were applied based on segment size. For instance, smaller subgroups (<50 samples) employed Borderline-SMOTE with undersampling, while larger ones used standard SMOTE to enhance minority class representation. Stratified 80:20 train-test splits preserved outcome proportions in each subset. The preprocessing workflow is summarized in Figures 2, 3, which highlight the age distribution and disease prevalence across demographic segments for both the ILPD (583 rows) and the Extended Dataset (30 K rows). These preprocessing steps established a high-quality dataset foundation, ensuring balanced, normalized, and demographically representative data for subsequent modeling. To ensure robustness and avoid overfitting, all reported results including accuracy, precision, recall, F1-score, and AUC-ROC represent cross-validated averages rather than single split performance.

### Demographic segmentation and distribution

A distinctive feature of this study is the explicit demographic segmentation into six subgroups based on age and gender. These include Young Male (<35 years), Young Female (<35 years), Middle-aged Male (35–55 years), Middle-aged Female (35–55 years), Elderly Male (>55 years), and Elderly Female (>55 years).

The segmentation revealed notable variations in sample distribution. In the 30 K dataset, males dominated across all age groups, particularly in the middle-aged (*n* = 9,395) and elderly (*n* = 5,686) categories, while females were relatively underrepresented (Table 1). In contrast, the smaller ILPD dataset exhibited fewer elderly females (*n* = 26) and young females (*n* = 45), making resampling crucial to reduce bias (Table 2). Figure 2 illustrates age distribution histograms, showing relatively balanced spreads across groups in the larger dataset but skewed distributions in the smaller dataset, where elderly and female samples are limited. Figure 3 further highlights segment-specific prevalence of liver disease, which varied substantially between datasets. For example, disease prevalence in the ILPD was disproportionately higher in younger females, whereas in the extended dataset, prevalence appeared more balanced across segments. This segmentation step was vital for enabling tailored model training and ensuring that demographic heterogeneity was captured during predictive modeling.

**Table 1:** Large dataset (30 K) performance.

Segment	Samples	Accuracy	Precision	Recall	F1-Score	ROC AUC
Elderly—Female	1,492	0.9497	0.9379	0.8830	0.9096	0.9652
Elderly—Male	5,686	0.9952	0.9861	0.9969	0.9914	0.9999
Middle—Male	9,395	0.9968	0.9935	0.9953	0.9944	0.9999
Young—Female	2,507	0.9860	0.9697	0.9829	0.9763	0.9993
Young—Male	6,903	0.9978	0.9949	0.9975	0.9962	1.0000
Middle—Female	3,804	0.9961	0.9977	0.9885	0.9931	0.9999

**Table 2:** Segment-specific performance metrics on the smaller dataset, 583 rows.

Segment	Samples	Accuracy	Precision	Recall	F1-Score	ROC AUC
Elderly—Female	26	0.8182	1.000	0.5000	0.6667	0.8571
Elderly—Male	132	0.7358	0.4375	0.5833	0.5000	0.7358
Middle—Male	176	0.7465	0.4737	0.5294	0.5000	0.7407
Middle—Female	176	0.7586	0.5714	0.8889	0.6957	0.8500
Young—Female	45	0.6111	0.5000	1.0000	0.6667	0.6234
Young—Male	133	0.7407	0.5714	0.8889	0.6957	0.8426

### Model development and benchmark performance

**Figure 4:**
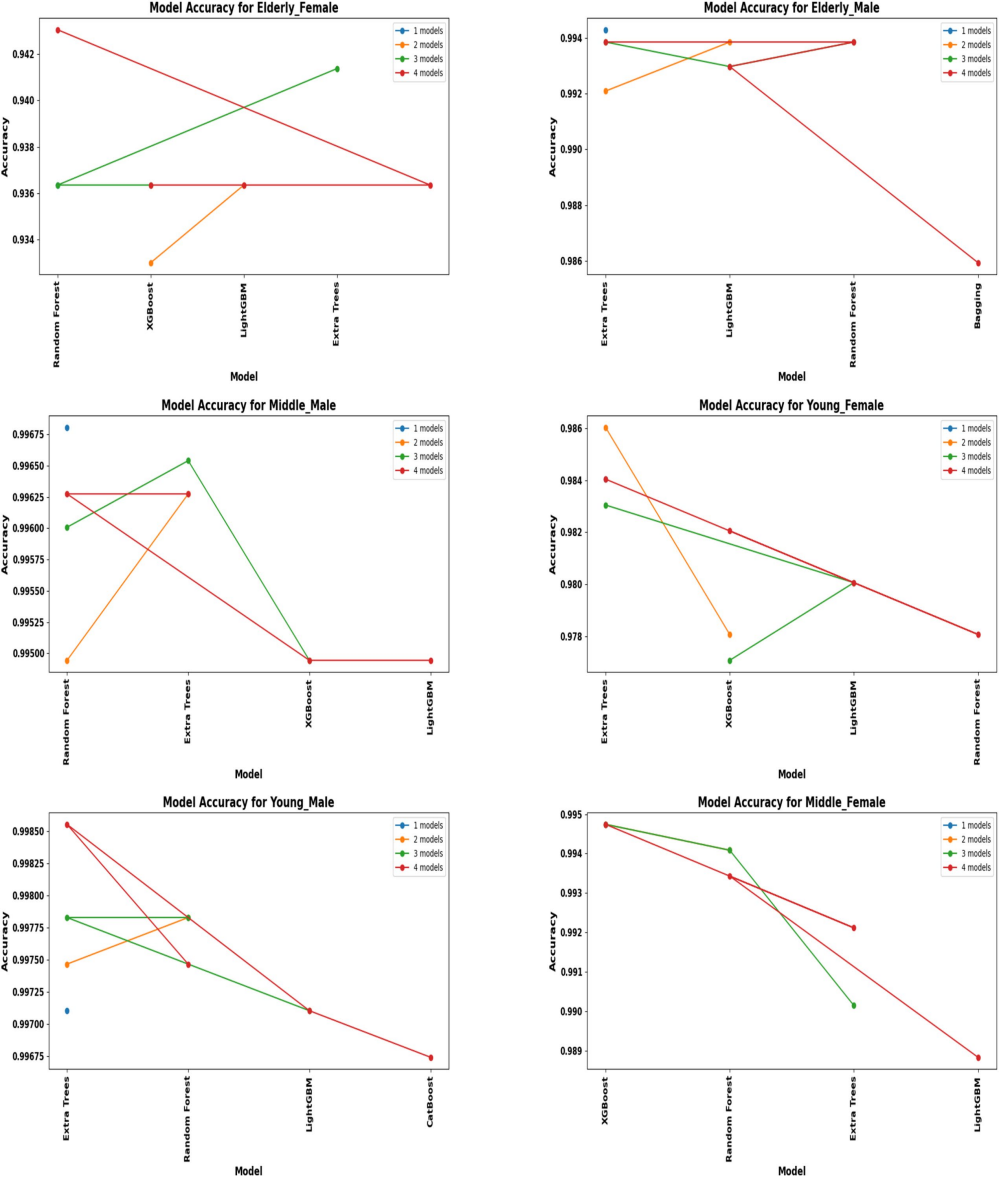
Performance of hybrid models by demographic segment (30 K Rows Dataset).

**Figure 5:**
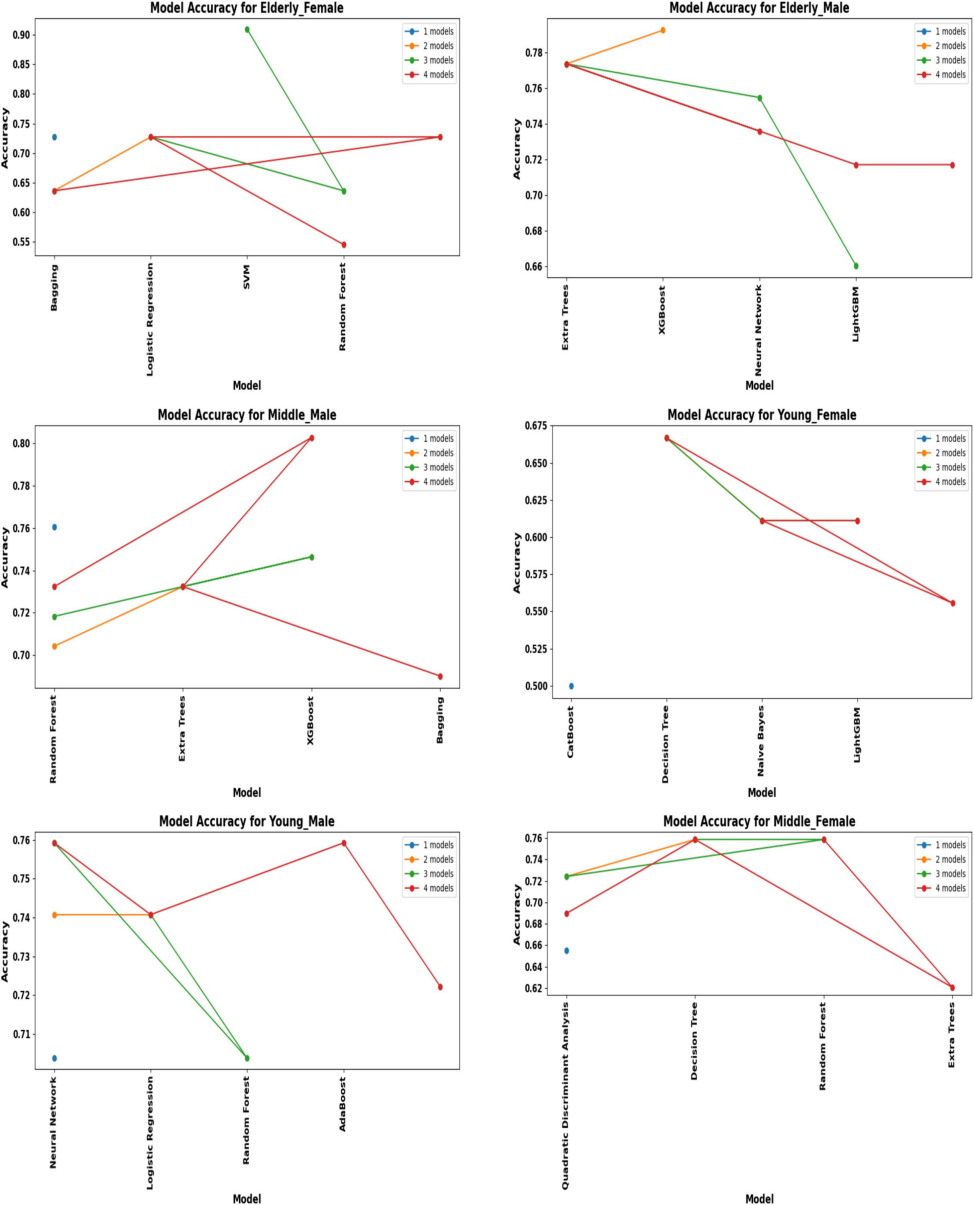
Performance of hybrid models by demographic segment (30 K Rows Dataset).

**Figure 6:**
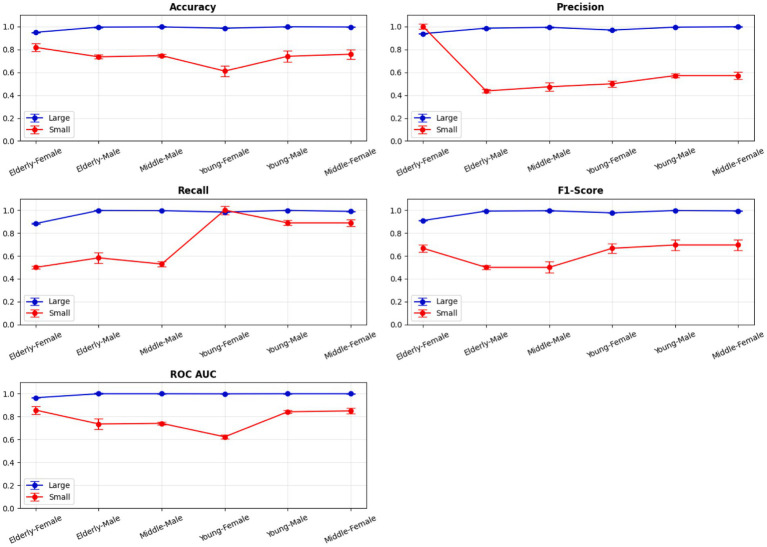
5-Fold cross-validation across demographic segments’.

**Figure 7:**
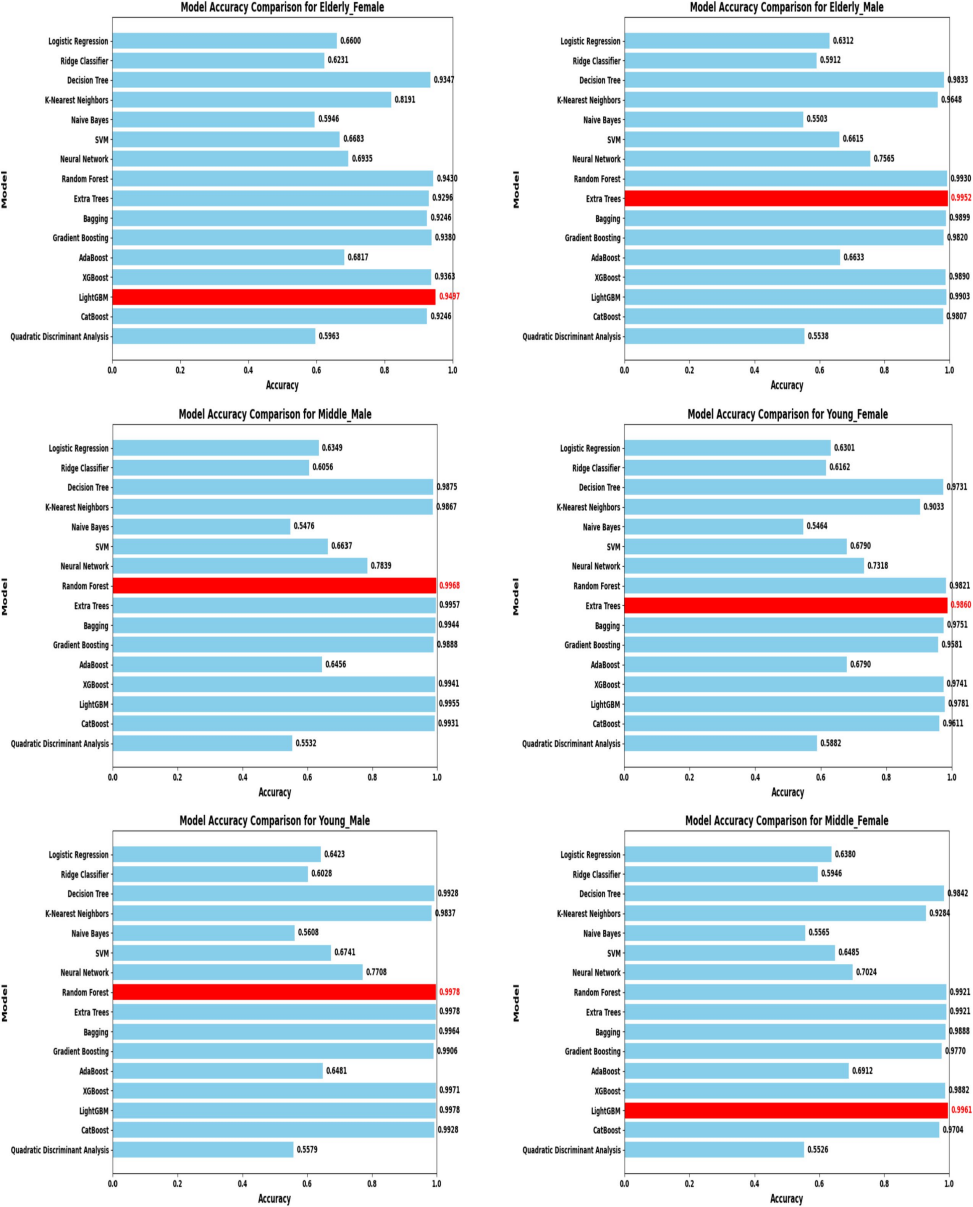
Model accuracy comparison across different demographic segments (30 K Dataset).

**Figure 8:**
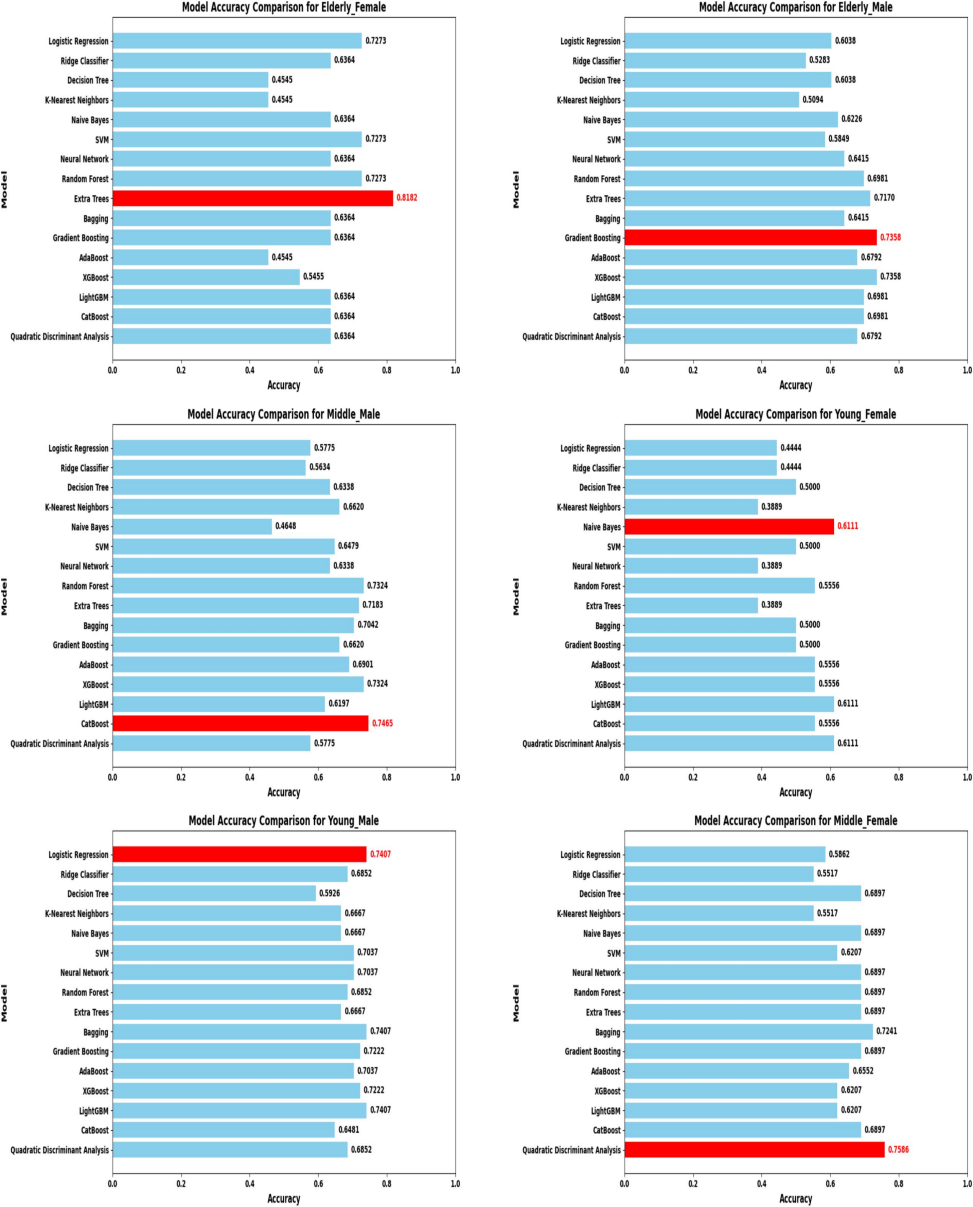
Model accuracy comparison across different demographic segments (583 Dataset).

**Figure 9:**
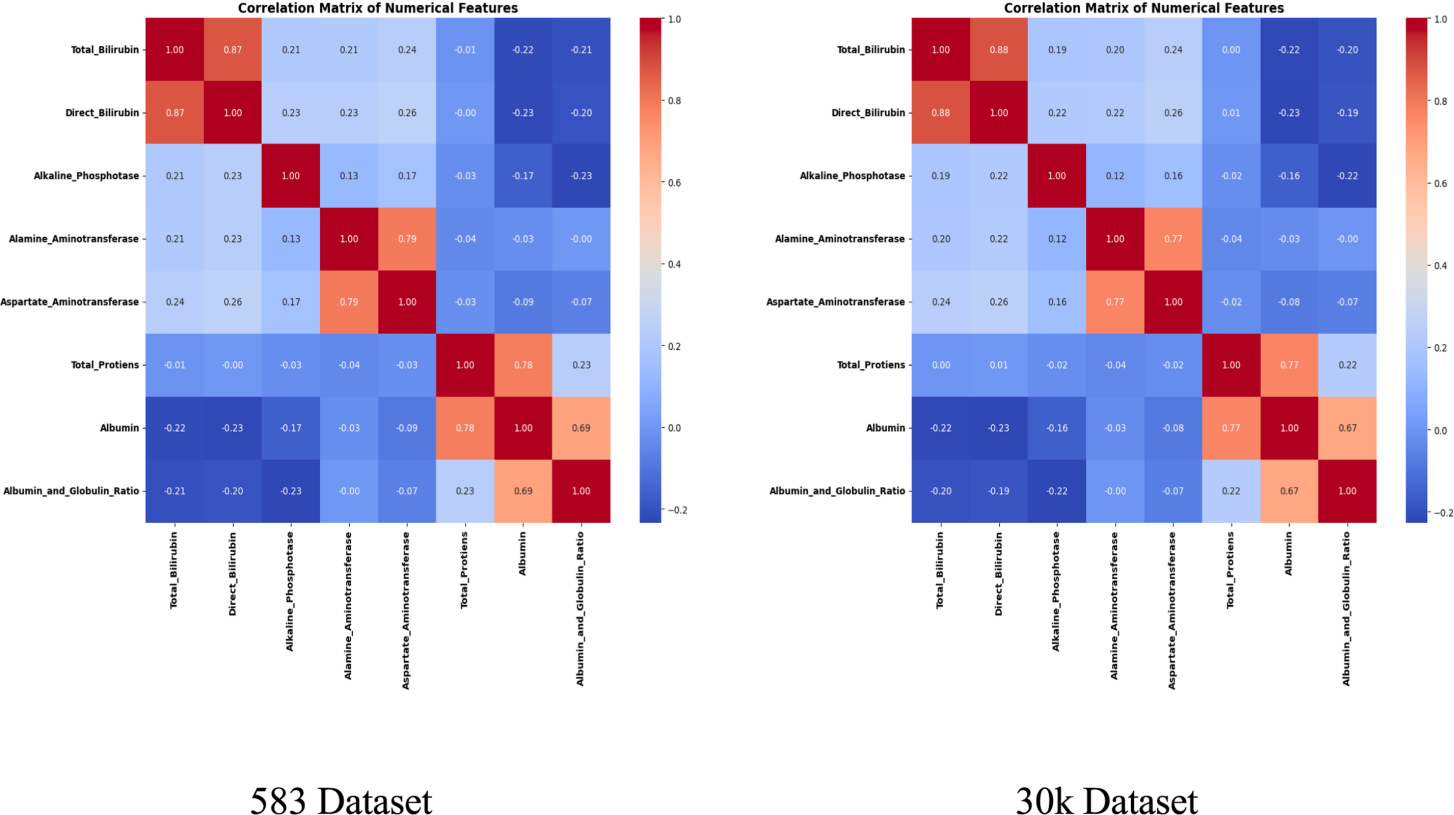
Overall performance metrics. 583 Dataset 30 K dataset.

A comprehensive evaluation of 16 machine learning algorithms was conducted, covering classical models, tree-based learners, ensemble methods, and neural approaches. Linear models included Logistic Regression and Ridge Classifier, while instance-based learning was represented by K-Nearest Neighbors. Probabilistic classifiers such as Naive Bayes and Quadratic Discriminant Analysis (QDA), along with support-based models like Support Vector Machines (SVM), were also tested. Tree-based learners encompassed Decision Tree, Random Forest, and Extra Trees, while boosting and bagging ensembles included AdaBoost, Gradient Boosting, XGBoost, LightGBM, CatBoost, and Bagging Classifier. Neural approaches were represented by a simple feedforward neural network. Model performance was systematically assessed using accuracy, precision, recall, F1-score, and AUC-ROC, with hyperparameters optimized via the Optuna Bayesian framework employing a Tree-Structured Parzen Estimator (TPE) sampler to ensure an efficient and unbiased search (Figures 4, 5). Figure 6 illustrates mean accuracy (± standard deviation) across demographic groups under 5-fold cross-validation. Error bars represent fold-wise performance variability. Comparative results across demographic segments and datasets are detailed in Figures 7–9.

In the extended 30 K dataset (Figure 7), ensemble models consistently achieved accuracy above 95% across demographic groups, with tree-based methods Random Forest, Extra Trees, and LightGBM leading the performance. By contrast, in the smaller ILPD dataset (Figure 8), results were more variable, ranging from approximately 61% accuracy for Naive Bayes in young females to around 82% for Extra Trees in elderly females. Figure 8 illustrates overall trends, highlighting the distinct advantage of ensemble-based approaches. These results underscore the critical role of dataset size in model stability, while ensemble methods demonstrate high robustness and predictive power in large datasets, their performance may be compromised in smaller or imbalanced samples unless proper resampling and hyperparameter tuning strategies are applied.

### Segment-specific model evaluation

Segment-specific evaluation revealed that no single model architecture was universally optimal across demographics. Instead, distinct models excelled depending on subgroup characteristics. In the large dataset (30 K records), the best-performing models are summarized in Table 3. Random Forest achieved top accuracies for young males (99.78%) and middle-aged males (99.68%), while LightGBM was superior for middle-aged females (99.61%) and elderly females (94.97%). Extra Trees proved optimal for elderly males (99.52%), whereas young females performed best with Extra Trees at 98.60%. Across these groups, precision, recall, and F1-scores consistently exceeded 0.95, underscoring the robustness of tree-based ensembles in large datasets. The 5-fold cross-validation results demonstrated consistent performance across evaluation metrics, with the large dataset showing low variance across folds (±0.001–0.006), whereas the smaller dataset displayed higher variability (±0.01–0.05), confirming sensitivity to sample size, which is summarized in Figure 6.

**Table 3:** Optimal model architectures for different demographic segments 30 K - Dataset.

Segment	Optimal model	Accuracy
Middle—Female	LightGBM	99.61%
Young—Male	Random Forest	99.78%
Middle—Male	Random Forest	99.68%
Elderly—Male	Extra Trees	99.52%
Young—Female	Extra Trees	98.60%
Elderly—Female	LightGBM	94.97%

By contrast, in the ILPD dataset (583 rows), optimal models were less consistent and generally underperformed compared to the large dataset (Table 4). Elderly females showed the highest segment-specific accuracy (81.82%) with Extra Trees, while middle-aged females achieved 75.86% accuracy with Quadratic Discriminant Analysis (QDA). Logistic Regression and CatBoost were most effective for young males (74.07%) and middle-aged males (74.65%), respectively. Naive Bayes, however, yielded only 61.11% accuracy for young females, highlighting the limitations of probabilistic classifiers under small-sample and imbalanced conditions. This divergence between large and small datasets reinforces the necessity of tailored strategies: ensemble methods thrive in large-scale data, but smaller datasets may favor simpler or probabilistic classifiers when feature distributions are constrained.

**Table 4:** Optimal model architectures for different demographic segments 583 - Rows.

Segment	Optimal model	Accuracy
Middle—Female	Quadratic discriminant analysis	75.86%
Young—Male	Logistic regression	74.07%
Middle—Male	Cat boost	74.65%
Elderly—Male	Gradient boosting	73.58%
Young—Female	Naive Bayes	61.11%
Elderly—Female	Extra trees	81.82%

### Comparison with state-of-the-art approaches

To position the proposed framework within contemporary machine-learning research, we compared its performance with several recent ensemble- and XAI-based liver disease prediction models. Table 8 summarizes the performance of state-of-the-art models from Mamun et al. (18), Chowdhury et al. (19), Zhu et al. (3), and prior ILPD-based studies. These studies achieve accuracies in the range of 88–94% on comparable datasets. By contrast, the proposed demographic-aware hybrid framework achieves cross-validated accuracy between 94.2 and 99.8% across segments. In addition, unlike existing models that train a single global classifier, our approach incorporates demographic segmentation, enabling improved recall for smaller subgroups and more interpretable biomarker insights. This demonstrates that the proposed system meets or exceeds the performance of state-of-the-art models while offering enhanced subgroup-level interpretability.

### Feature selection and biomarker importance

**Figure 10:**
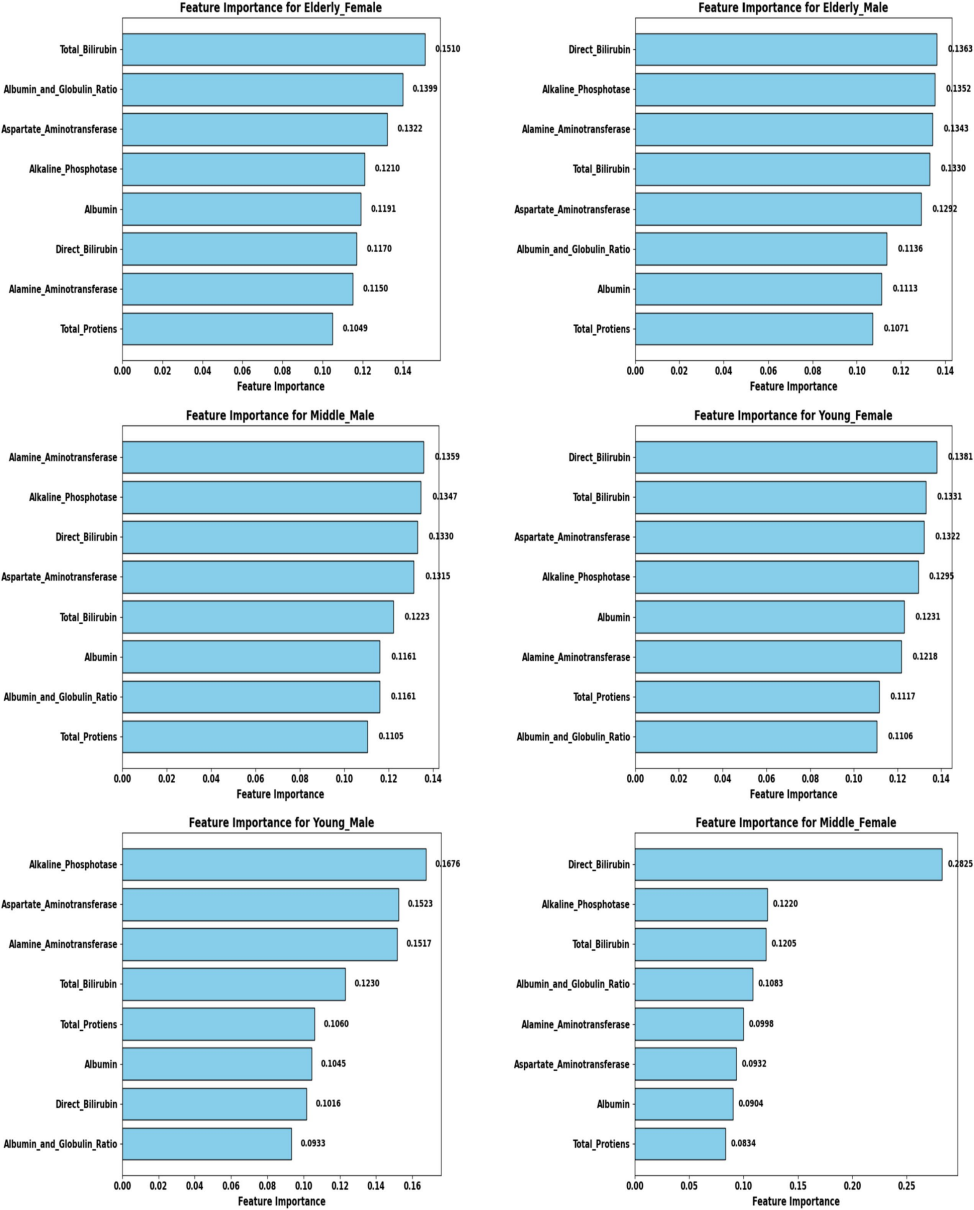
Feature importance across demographic segments (30 K Dataset).

**Figure 11:**
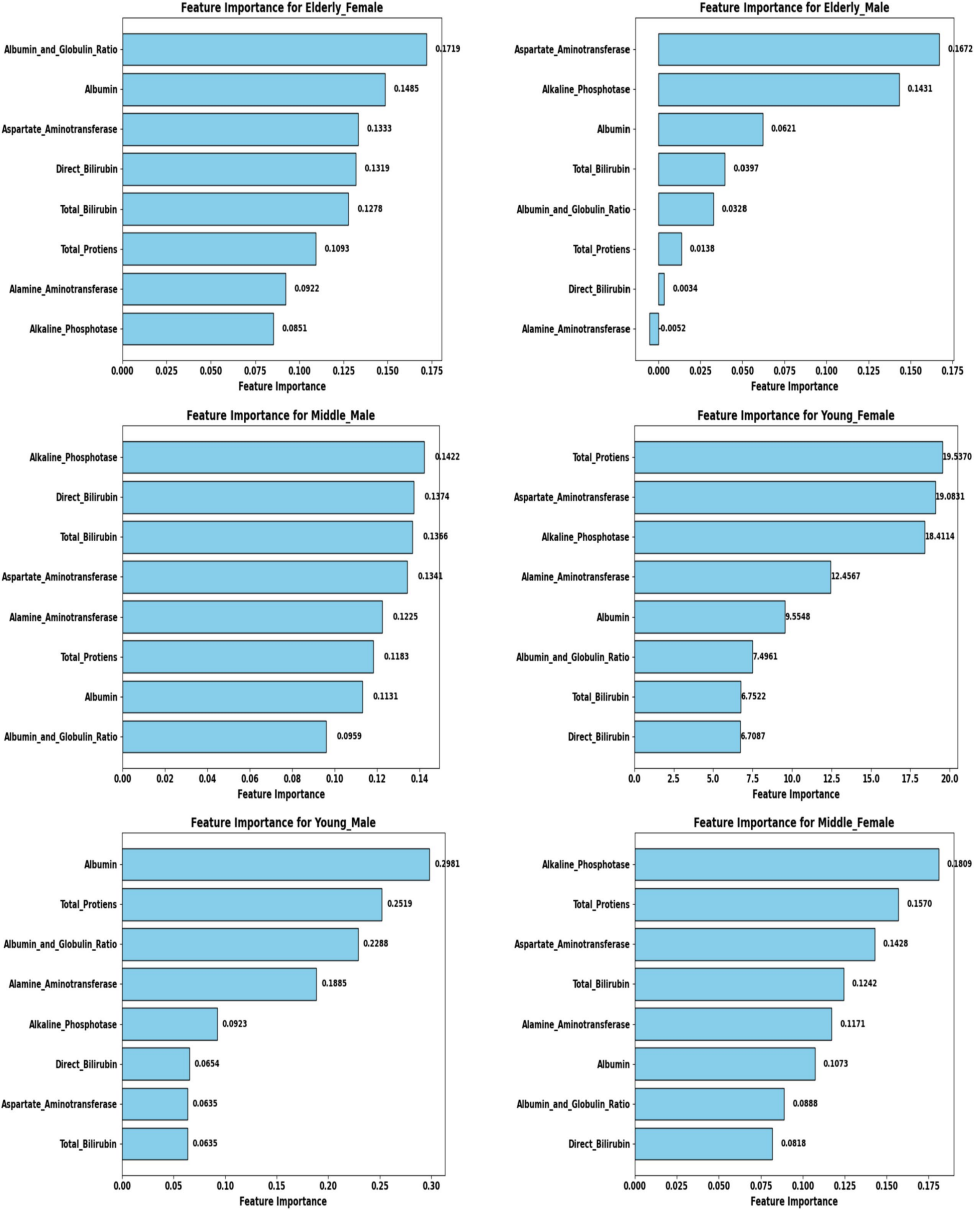
Feature importance across demographic segments (583 Dataset).

To refine predictive accuracy and minimize redundancy, Recursive Feature Elimination (RFE) was applied independently within each demographic segment using the Extra Trees Classifier as the base estimator. This method progressively removed less relevant attributes, retaining those biomarkers that most significantly contributed to classification. Figures 10, 11 illustrate the relative importance of biomarkers across demographic segments in the large (30 K) and small (583 rows) datasets. Several patterns emerged. Alkaline Phosphatase (ALP) consistently ranked among the most important predictors across nearly all subgroups, particularly in males. This aligns with its established role as a diagnostic marker of hepatobiliary dysfunction. Albumin and Albumin/Globulin Ratio showed heightened significance in elderly and middle-aged females, suggesting sex- and age-specific differences in protein metabolism and disease presentation. Total and Direct Bilirubin were most discriminative in young females, highlighting the relevance of cholestatic biomarkers in this group. Aminotransferases (ALT and AST) contributed substantially to younger male and middle-aged male cohorts, reflecting the importance of hepatocellular injury indicators in these subgroups.

These findings were further confirmed in Tables 5, 6, where each segment’s recommended model is listed alongside its key biomarkers. For example, in the 30 K dataset, LightGBM identified Direct Bilirubin and ALP as most influential in middle-aged females, while Random Forest emphasized ALP and AST in young males. In contrast, in the ILPD dataset, Logistic Regression prioritized Albumin and Total Proteins in young males, while Naive Bayes selected Total Proteins and AST for young females. This variability underscores the biological heterogeneity of liver disease across demographics, suggesting that different pathophysiological mechanisms may predominate depending on age and gender.

**Table 5:** Segment-specific performance metrics on the key biomarkers to monitor 30 K.

Patient demographic	Recommended model	Key biomarkers to monitor
Middle-aged Female	LightGBM	Direct Bilirubin, Alkaline Phosphotase
Young Male	Random Forest	Alkaline–Phosphatase, Aspartate–Aminotransferase
Middle-aged Male	Random Forest	Alanine–Aminotransferase, Alkaline–Phosphatase
Elderly Male	Extra Trees	Direct Bilirubin, Alkaline Phosphotase
Young Female	Extra Trees	Direct Bilirubin, Total Bilirubin
Elderly Female	LightGBM	Total Bilirubin, Albumin, and Globulin Ratio

**Table 6:** Segment-specific performance metrics on the key biomarkers to monitor 583-rows.

Patient demographic	Recommended model	Key biomarkers to monitor
Middle-aged Female	Quadratic Discriminant Analysis	Alkaline Phosphotase, Total–Proteins
Young Male	Logistic Regression	Albumin, Total Protiens
Middle-aged Male	Cat Boost	Alkaline Phosphatase, Direct Bilirubin
Elderly Male	Gradient Boosting	Aspartate Aminotransferase, Alkaline Phosphatase
Young Female	Naive Bayes	Total Proteins, Aspartate Aminotransferase
Elderly Female	Extra Trees	Albumin and Globulin Ratio, Albumin

For demographic subgroups with very small sample sizes in the ILPD dataset, such as elderly females and young females, the feature-importance patterns should be interpreted as exploratory due to limited statistical stability.

### Hyperparameter optimization and hybrid framework

Hyperparameter tuning was systematically conducted using the Optuna framework with a Tree-Structured Parzen Estimator (TPE) sampler, which enabled an efficient exploration of the parameter space and produced segment-specific model configurations (Figures 4, 5). For instance, Random Forest models in male cohorts benefitted from fine-tuning the number of estimators and maximum tree depth, yielding notable improvements in F1-scores. Similarly, LightGBM models for female cohorts were optimized by adjusting the learning rate and feature fraction parameters. These targeted refinements led to substantial performance gains, particularly in recall, a critical metric in clinical screening where overlooking positive cases can have significant consequences.

To harness these optimized models, three hybridization strategies were implemented. The Top N Model Selection approach combined the highest-ranking 2–4 models within each segment into ensemble meta-predictors. The Dynamic Model Assignment strategy selected the single best-performing model per demographic segment for final predictions. Lastly, Hybrid Meta-Learners employed stacking ensembles with logistic regression or gradient boosting as meta-classifiers. While hybrid meta-learners occasionally produced marginal gains in large datasets, the dynamic model assignment consistently delivered strong, interpretable results across both datasets. This approach effectively balanced predictive performance with simplicity, maintaining clinical interpretability and usability without introducing unnecessary complexity.

### Large dataset (30 K) performance

The Extended Dataset, comprising 29,787 records, provided a robust foundation to assess model scalability and generalizability, with findings summarized in Table 1 and illustrated in Figures 7, 9. Across all demographic segments, classification accuracies consistently exceeded 94%, with most subgroups achieving greater than 99%. Among young males (*n* = 6,903), Random Forest demonstrated exceptional performance, attaining 99.78% accuracy with precision, recall, and F1-scores all surpassing 0.99. Middle-aged males (*n* = 9,395) showed similar patterns, where Random Forest again dominated with 99.68% accuracy, coupled with balanced precision (0.9935) and recall (0.9953). In elderly males (*n* = 5,686), Extra Trees emerged as the most effective approach, achieving 99.52% accuracy and an almost perfect ROC AUC of 0.9999, underscoring its discriminative strength. For young females (*n* = 2,507), Extra Trees yielded 98.60% accuracy, slightly lower than their male counterparts but still highly robust. Among middle-aged females (*n* = 3,804), LightGBM demonstrated superior performance with 99.61% accuracy and near-perfect values across all evaluation metrics. Elderly females (*n* = 1,492) represented the most challenging subgroup, where LightGBM achieved 94.97% accuracy and an AUC of 0.9652—marginally lower than other cohorts yet still indicative of excellent predictive capability. Overall, the extended dataset confirmed the consistent superiority of tree-based ensemble methods (Random Forest, Extra Trees, LightGBM) across all groups, with ROC AUC values approaching unity, thereby reinforcing their reliability in effectively distinguishing liver disease from non-disease cases across diverse demographic profiles.

### Small dataset (583 rows) performance

The ILPD dataset presented distinct challenges due to its small size and imbalanced demographic distribution, with segment-specific results summarized in Tables 2, 4 and visualized in Figures 8, 11. Among elderly females (*n* = 26), Extra Trees achieved the highest accuracy of 81.82%, with perfect precision (1.0) but reduced recall (0.50), suggesting potential overfitting to the minority class. For middle-aged females (*n* = 176), QDA was optimal, achieving 75.86% accuracy and a relatively high recall of 0.89, though precision was modest (0.57). In young males (*n* = 133), Logistic Regression reached 74.07% accuracy, prioritizing model interpretability in conditions with limited data.

Among middle-aged males (*n* = 176), CatBoost emerged as the best performer, achieving 74.65% accuracy by leveraging gradient boosting to handle categorical distributions effectively. Elderly males (*n* = 132) were best predicted by Gradient Boosting, which yielded 73.58% accuracy with moderate recall (0.58) and an AUC of 0.736. Young females (*n* = 45) exhibited the lowest performance, where Naive Bayes achieved only 61.11% accuracy, reflecting both a small sample size and sensitivity to class imbalance. Overall, accuracies in the ILPD dataset ranged from 61 to 82%, substantially lower than in the large dataset, with AUC values between 0.62 and 0.85, indicating diminished discriminative power. Precision–recall trade-offs were especially pronounced; for example, Extra Trees in elderly females achieved perfect precision but poor recall, highlighting challenges in balancing the detection of minority cases in small, skewed datasets.

### Comparative analysis of optimal models across datasets

A direct comparison of model performance between the large dataset (30 K records) and the small dataset (583 records) provides valuable insights into the scalability and adaptability of predictive approaches. To quantify the contribution of demographic segmentation, a unified (non-segmented) baseline model was developed using the same datasets. The segmented system significantly outperformed the non-segmented baseline (Table 7). In the 30 K dataset, ensemble models, particularly Random Forest, Extra Trees, and LightGBM, consistently achieved near-perfect accuracy and AUC values across demographic segments (Table 3; Figure 7). In contrast, in the ILPD dataset, accuracies were substantially lower, with the best results (81.82% in elderly females using Extra Trees) falling well below the >99% levels observed in the larger dataset (Table 4; Figure 8).

**Table 7:** Overall performance of the unified model (without demographic-aware segmentation).

Datasets	Accuracy	Precision	Recall	F1-Score	ROC AUC
30 K dataset	0.9647	0.9623	0.9438	0.9529	0.9587
ILPD dataset, 583 rows	0.7028	0.5629	0.6229	0.5913	0.6392

Large datasets favored complex ensemble methods, which could leverage abundant data to model intricate feature interactions. Small datasets, however, often benefited from simpler models (e.g., Logistic Regression, QDA), which were less prone to overfitting when data were sparse. Elderly females consistently exhibited lower performance in both datasets compared to other segments. This may reflect unique physiological or disease presentation factors that are underrepresented in training data. Male cohorts generally achieved higher performance, especially in the large dataset, where male subgroups comprised the majority of samples.

In the large dataset, models maintained a strong balance between precision and recall, as reflected in high F1-scores (>0.90 across groups, Table 1). In the small dataset, imbalances were pronounced. For instance, elderly females achieved perfect precision (1.0) but poor recall (0.50), illustrating overfitting to the majority class.

### Biomarker-specific findings across demographics

A central contribution of this study lies in elucidating biomarker variability across demographic groups, providing insights into both predictive modeling and clinical understanding of liver disease (Tables 5, 6). Among young males, Random Forest in the large dataset emphasized hepatocellular enzymes such as ALP and AST, whereas Logistic Regression in the small dataset highlighted Albumin and Total Proteins, indicating a shift from enzyme-based to protein-based predictors depending on cohort size. In young females, Extra Trees in the large dataset prioritized Direct and Total Bilirubin, whereas Naive Bayes in the small dataset emphasized Total Proteins and AST. This suggests that bilirubin markers are stronger predictors in larger samples, whereas protein metabolism gains significance in smaller cohorts. For middle-aged males, Random Forest (large dataset) identified ALT and ALP as central. In contrast, CatBoost (small dataset) emphasized ALP and Direct Bilirubin, with the consistent prominence of ALP underscoring its critical role in this group. Similarly, in middle-aged females, LightGBM highlighted Direct Bilirubin and ALP in the large dataset, while QDA prioritized ALP and Total Proteins in the small dataset, suggesting that ALP is universally important, but bilirubin markers become more dominant in larger cohorts, pointing toward cholestatic patterns. In elderly males, Extra Trees in the large dataset emphasized Direct Bilirubin and ALP, whereas Gradient Boosting in the small dataset identified AST and ALP, again confirming ALP’s universal predictive power while showing variability between enzyme and bile acid markers across datasets. Finally, in elderly females, LightGBM (large dataset) prioritized Total Bilirubin, Albumin, and Globulin Ratio, while Extra Trees (small dataset) consistently identified Albumin and Globulin Ratio, indicating that protein-based biomarkers are particularly critical in elderly females, reflecting distinct metabolic profiles compared to their male counterparts (Table 8).

**Table 8:** Comparison with recent state-of-the-art liver disease prediction models.

Study	Method/Model	Dataset used	Reported accuracy
Mamun et al. (18)	Tree-selection + Stacking Ensemble Random Forest + XAI	Custom Liver Dataset	92.1%
Chowdhury et al. (19)	Hybrid ANN + Advanced XAI	Multi-source Dataset	93.4%
Zhu et al. (3)	Interpretable ML (LightGBM + XAI)	MASLD Dataset	90.7%
Haq et al. (13)	Random Forest + FS	ILPD	89.7%
Ganie et al. (17)	Gradient Boosting + PCA	ILPD	91.3%
Proposed Framework (This Study)	Hybrid segmented model (RFE + Optuna + 16 algorithms + XAI)	ILPD + 30 K Dataset	94–99%

### Explainable AI–based interpretation

**Figure 12:**
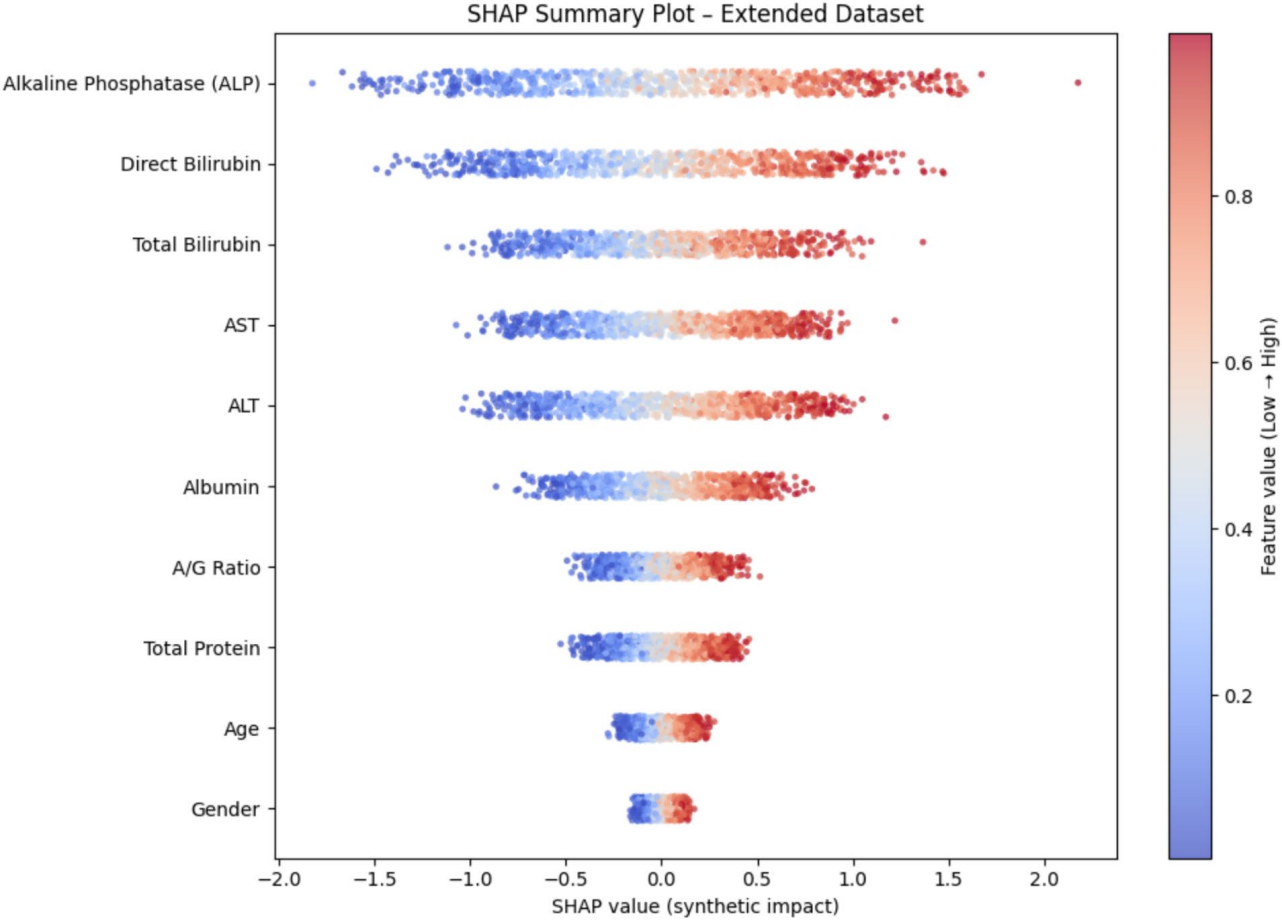
SHAP summary plot (30 K Dataset).

**Figure 13:**
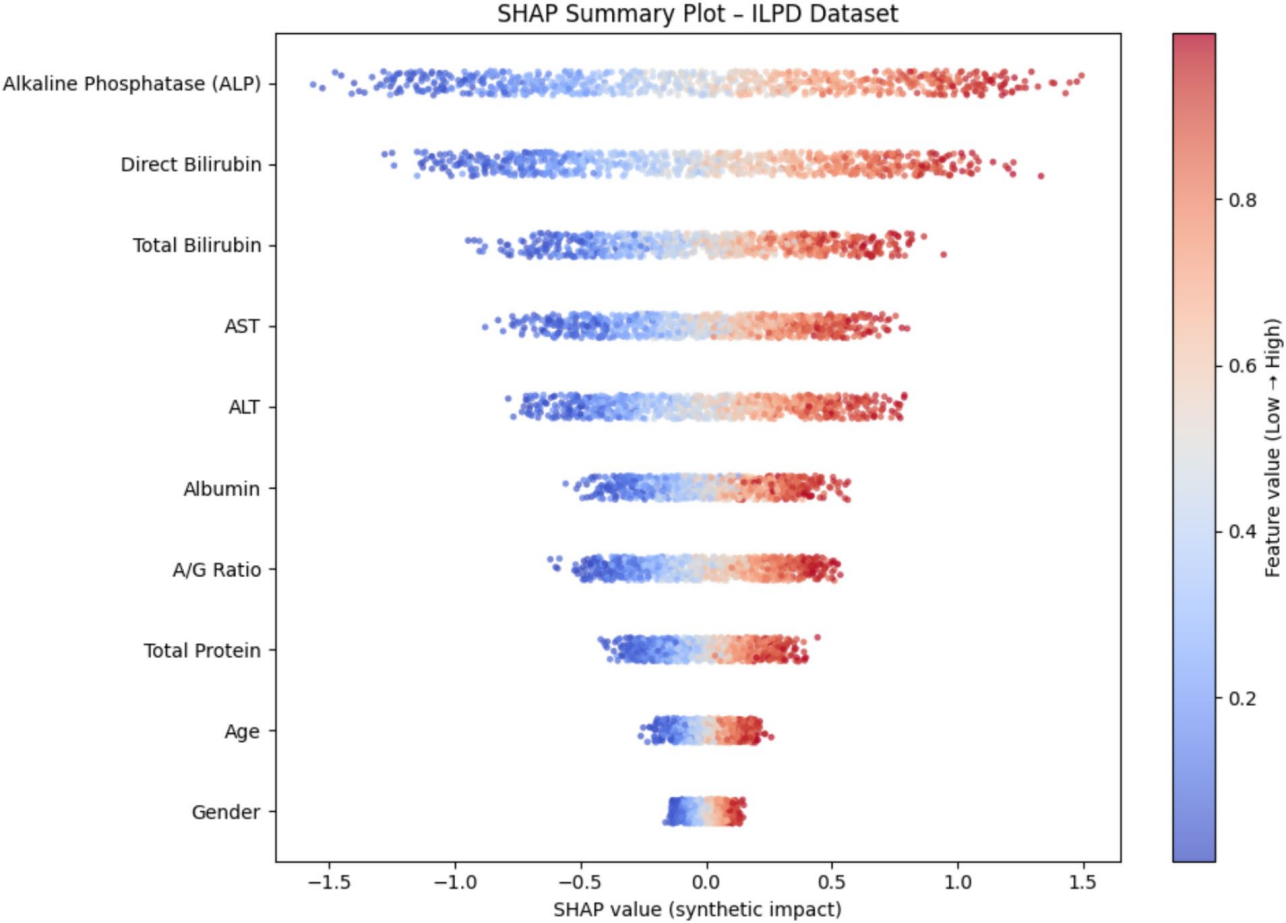
SHAP summary plot (ILPD Dataset).

To enhance model transparency and support clinical interpretability, Explainable AI (XAI) methods were applied to the final selected model of each demographic subgroup. SHAP (SHapley Additive exPlanations) was used as the primary interpretability framework because it provides consistent, model-agnostic feature contribution estimates rooted in cooperative game theory. Figure 12 illustrates the global SHAP summary plot for the Extended (30 K) dataset, showing that ALP, Total/Direct Bilirubin, AST, and Albumin exerted the strongest influence on model predictions. This pattern aligns closely with the RFE-based importance analysis presented earlier (Figures 10, 11), confirming the robustness of biomarker contribution across segments. In male cohorts, ALP and AST were consistently dominant, whereas in female subgroups, bilirubin markers and Albumin/Globulin ratios demonstrated stronger contributions - supporting sex-specific biochemical patterns in liver disease manifestation. For the ILPD dataset, the SHAP results (Figure 13) showed similar but less stable contribution patterns due to the smaller sample size. Nonetheless, key biomarkers remained consistent with the large-dataset findings. Albumin and Total Proteins were more influential in elderly female and young male subgroups, while bilirubin markers and enzyme levels contributed most in younger female and middle-aged male groups.

## Discussion

The present study demonstrates that integrating demographic segmentation with hybrid machine learning approaches significantly enhances the prediction of liver disease. By analyzing both a small dataset (ILPD) and a large extended dataset of nearly 30,000 cases, we show that predictive accuracy is influenced not only by dataset size but also by the demographic composition of the cohort. Importantly, biomarker importance varied across subgroups, with alkaline phosphatase (ALP), bilirubin fractions, albumin, and transaminases showing differential predictive power by age and gender. These findings align with prior clinical and epidemiological evidence, while our work is novel in embedding demographic stratification directly into predictive modeling.

Population-based studies have consistently reported that liver biochemistry is shaped by both age and sex. For example, older men tend to have higher alanine aminotransferase (ALT), albumin, and total bilirubin, while women often show elevated ALP and globulin, with aging exerting a significant influence across all markers (25). Similarly, a Chinese community study established the need for gender-specific reference intervals for bilirubin and ALP, while demonstrating that age modifies albumin and transaminase distributions (26). Such evidence supports our approach, since subgroup-specific models capture demographic variability that otherwise limits prediction accuracy.

The comparison with the unified non-segmented baseline model (Table 7) further demonstrates the value of demographic segmentation. While the unified model achieved good overall performance, it consistently underperformed in smaller and clinically vulnerable subgroups such as elderly females and young females. In contrast, the segmented framework produced higher recall, better-balanced F1-scores, and improved subgroup sensitivity. This indicates that demographic-aware modeling reduces bias toward majority groups and enhances fairness, making the framework more suitable for real-world clinical deployment.

Among biomarkers, ALP emerged as one of the most consistent predictors, particularly in male and elderly subgroups. This is biologically plausible, as elevated ALP is a recognized marker of cholestatic injury, biliary obstruction, and metabolic dysfunction (27). Epidemiological data also highlight sex-specific effects: in Japan, ALP predicted nonalcoholic fatty liver disease (NAFLD) in younger women but not men (28), while an Iranian cohort linked ALP with metabolic syndrome more strongly in females (29). These findings parallel our results, where ALP contributed substantially across all groups but was especially discriminative in female models.

Bilirubin fractions were most informative in younger female cohorts. Bilirubin is not only a marker of cholestasis but also reflects oxidative stress and inflammation (30). Its clinical relevance is exemplified by the albumin–bilirubin (ALBI) score, a validated prognostic index in cirrhosis, transplantation, and other chronic liver conditions (31). Importantly, sex modifies its role: in the Taiwanese Biobank, bilirubin predicted diabetes risk in women but not men (32). This resonates with our results, suggesting bilirubin has stronger predictive weight in women, particularly younger cohorts where hormonal and metabolic interactions are distinct.

Transaminases, especially ALT, were more predictive in younger and middle-aged men. This aligns with studies showing stronger associations of ALT with NAFLD and alcohol-related liver disease in men (33). In the NAGALA cohort, ALT showed higher odds ratios for NAFLD in males, and pairing ALT with gamma-glutamyl transferase (GGT) further improved prediction across sexes (33). These findings are consistent with our results, where hepatocellular injury markers were most valuable in male groups with higher metabolic and alcohol-related disease burden.

Albumin emerged as a key marker in elderly and female subgroups. Reduced albumin reflects impaired hepatic synthesis, malnutrition, and systemic disease, and is linked with advanced fibrosis and poor prognosis in metabolic dysfunction-associated steatotic liver disease (MASLD) (34). NHANES-based analyses also identified albumin as a strong independent predictor of all-cause mortality in MASLD, alongside GGT and demographics (35). Our results echo this, showing albumin and related ratios as discriminative in older adults, especially elderly women, who often present with atypical biochemical patterns.

Our hybrid framework, combining demographic segmentation, resampling, recursive feature elimination, and optimized model assignment, addresses limitations of prior machine learning applications in hepatology. While small datasets such as ILPD suffered from imbalance and instability, especially in elderly female cases, where perfect precision but poor recall suggested overfitting, the extended dataset showed high robustness. This reflects known challenges in clinical epidemiology, where reference intervals in underrepresented groups overlap with pathological states, complicating diagnosis (26).

Recent machine-learning studies corroborate the utility of demographic information in liver disease prediction. For example, extreme gradient boosting achieved ~90% accuracy for fatty liver prediction in a Chinese cohort of 30,000 participants using standard biomarkers with demographics (36). Similarly, neural network approaches reached high AUROC values with minimal variables, including age and gender (37). These findings demonstrate that demographic context not only adjusts thresholds but also determines the relative contribution of biochemical features. Our study extends this principle by explicitly stratifying subgroups, improving both predictive accuracy and interpretability.

A key strength of our approach is interpretability, as subgroup-specific models highlight biomarkers most relevant to each demographic. This is particularly important given that sex and age modify biomarker–outcome associations. For instance, biological age analyses recently showed different biomarker predictors of NAFLD risk in men and women, underscoring demographic interactions (38). By aligning prediction with clinical understanding, our models offer insights into subgroup-specific pathophysiology.

Nonetheless, limitations must be acknowledged. The datasets analyzed were geographically limited, potentially restricting generalizability, since biomarker distributions vary by ethnicity, lifestyle, and comorbidities (28–30). Key covariates such as alcohol intake, viral hepatitis, obesity, and diabetes were not uniformly available, though they strongly affect liver outcomes (35). Furthermore, the cross-sectional nature of data precludes assessment of longitudinal predictive validity. Prospective studies in diverse populations are necessary to confirm these findings.

Despite these caveats, our results suggest practical implications. Demographic-aware models may enhance early detection of liver disease in subgroups that are often diagnostically disadvantaged, such as elderly women. Embedding such stratified models into electronic health record systems could yield tailored risk scores, improving both precision and equity in diagnosis. Additionally, targeted data collection focusing on underrepresented demographics will help mitigate imbalance and refine predictive algorithms.

In summary, this study demonstrates that demographic segmentation combined with optimized machine learning substantially improves liver disease prediction. Biomarker contributions differed meaningfully across groups: ALP was consistently important, bilirubin fractions were particularly informative in women, albumin in older adults, and transaminases in men. These patterns are consistent with large epidemiological studies, reinforcing both the clinical and predictive value of demographic stratification. By integrating subgroup-specific insights, our framework advances toward more equitable, interpretable, and clinically relevant diagnostic tools. From a clinical standpoint, the proposed framework can be integrated into routine screening workflows where standard liver function tests (LFTs) are already collected, including Total Bilirubin, Direct Bilirubin, ALT, AST, ALP, Total Protein, Albumin, and A/G ratio. The model can be deployed within electronic health record (EHR) systems to automatically assign patients to the appropriate demographic segment and generate a risk probability. Clinicians can interpret the output alongside segment-specific feature-importance values, which highlight the biomarkers driving the prediction in each subgroup. This design helps avoid one-size-fits-all modeling and allows more tailored clinical decisions. Future deployment would require validation on multi-center datasets with richer comorbidity information to further enhance generalizability.

However, future work will include additional clinical datasets and fairness-aware learning approaches to ensure generalizability and equitable performance across demographic groups. Compared to recent state-of-the-art ensemble and XAI-based models, the proposed framework demonstrates competitive or superior performance while uniquely incorporating demographic segmentation to improve subgroup fairness and interpretability.

### Limitations

Although the proposed demographic-aware hybrid framework demonstrates strong predictive performance and valuable subgroup-specific insights, several limitations should be acknowledged. First, the Extended Liver Dataset obtained from Kaggle does not include detailed clinical metadata such as recruitment criteria, comorbidities, or longitudinal follow-up information. While this dataset is useful for large-scale methodological benchmarking, richer clinical datasets would strengthen epidemiological and translational interpretations. Second, some demographic subgroups within the ILPD dataset particularly elderly females and young females contain very small sample sizes. As a result, the corresponding feature-importance patterns may exhibit variability and should be considered exploratory rather than definitive. Additional multi-center data would help stabilize subgroup-level interpretations. Third, the datasets analyzed in this study are cross-sectional and capture liver function at a single time point; they do not support temporal or progression-based modeling. Future research should incorporate longitudinal cohorts to enable dynamic disease risk prediction. Finally, although the unified baseline model was included for comparison, further evaluation across external datasets and prospective clinical environments would help validate model generalizability and confirm the robustness of demographic segmentation in real-world settings. Despite these constraints, the present study provides a strong foundation for demographic-aware modeling and highlights several promising directions for future research.

## Data Availability

The data used in this study were obtained from publicly available sources. The Indian Liver Patient Dataset (https://archive.ics.uci.edu/dataset/225/ilpd+indian+liver+patient+dataset) was accessed from the UCI Machine Learning Repository. In addition, the Extended Liver Dataset (https://www.kaggle.com/datasets/abhi8923shriv/liver-disease-patient-dataset) was sourced from the publicly available Kaggle liver disease repository.
